# Human Plasma Lipid Modulation in Schistosomiasis Mansoni Depends on Apolipoprotein E Polymorphism

**DOI:** 10.1371/journal.pone.0101964

**Published:** 2014-07-22

**Authors:** Caíque Silveira Martins da Fonseca, Adenor Almeida Pimenta Filho, Bianka Santana dos Santos, César Augusto da Silva, Ana Lúcia Coutinho Domingues, James Stuart Owen, Vera Lúcia de Menezes Lima

**Affiliations:** 1 Departamento de Bioquímica, Centro de Ciências Biológicas, Universidade Federal de Pernambuco (UFPE), Recife, Brazil; 2 Colegiado de Medicina, Universidade Federal do Vale do São Francisco (UNIVASF), Petrolina, Brazil; 3 Departamento de Medicina Clínica, Centro de Ciências da Saúde, Hospital das Clinicas, UFPE, Recife, Brazil; 4 Division of Medicine, University College London Medical School, Royal Free Campus, London, United Kingdom; Wayne State University, United States of America

## Abstract

**Background:**

Schistosomiasis mansoni is a parasitic liver disease, which causes several metabolic disturbances. Here, we evaluate the influence of Apolipoprotein E (*APOE*) gene polymorphism, a known modulator of lipid metabolism, on plasma lipid levels in patients with hepatosplenic schistosomiasis.

**Methodology/Principal Findings:**

Blood samples were used for *APOE* genotyping and to measure total cholesterol (TC), LDL-C, HDL-C and triglycerides. Schistosomiasis patients had reduced TC, LDL-C and triglycerides (25%, 38% and 32% lower, respectively; *P*<0.0001) compared to control individuals, whereas HDL-C was increased (10% higher; *P* = 0.0136). Frequency of the common alleles, ε2, ε3 and ε4, was similar (*P* = 0.3568) between controls (n = 108) and patients (n = 84), implying that *APOE* genotype did not affect susceptibility to the advanced stage of schistosomiasis. Nevertheless, while patient TC and LDL-C levels were significantly reduced for each allele (except TC in ε2 patients), changes in HDL-C and triglycerides were noted only for the less common ε2 and ε4 alleles. The most striking finding, however, was that accepted regulation of plasma lipid levels by *APOE* genotype was disrupted by schistosomiasis. Thus, while ε2 controls had higher TC and LDL-C than ε3 carriers, these parameters were lower in ε2 versus ε3 patients. Similarly, the inverse relationship of TG levels in controls (ε2>ε3>ε4) was absent in patients (ε2 or ε4>ε3), and the increase in HDL-C of ε2 or ε4 patients compared to ε3 patients was not seen in the control groups.

**Conclusion/Significance:**

We confirm that human schistosomiasis causes dyslipidemia and report for the first time that certain changes in plasma lipid and lipoprotein levels depend on *APOE* gene polymorphism. Importantly, we also concluded that *S. mansoni* disrupts the expected regulation of plasma lipids by the different ApoE isoforms. This finding suggests ways to identify new metabolic pathways affected by schistosomiasis and also potential molecular targets to treat associated morbidities.

## Introduction

Schistosomiasis, caused by *Schistosoma mansoni* worms, is one of the most prevalent parasitic diseases. More than 200 million people are infected and worldwide at least 280,000 people die because of schistosomiasis every year, most in developing countries [1], [2]. *S. mansoni* infections progress to hepatic fibrosis associated with portal blood hypertension [3] and 5–10% of patients present with the most severe form, hepatosplenic schistosomiasis [4], [5].

Previous studies have reported that human schistosomiasis alters plasma lipid composition [6]–[9] and metabolism [10]. From animal model studies, it is generally agreed that *S. mansoni* infection reduces levels of plasma cholesterol and triglycerides in both rodents [11], [12] and non-human primates [13], [14]. Nevertheless, the mechanisms behind these changes and the possible consequences for human health are not well understood.

One factor known to affect human plasma lipid concentrations is Apolipoprotein E (*APOE*, gene; ApoE, protein), which distributes between triglyceride-rich lipoproteins (very-low-density lipoproteins, VLDL and postprandial chylomicrons) and high-density lipoproteins (HDL), helping to regulate their metabolism and the plasma levels of cholesterol and triglyceride. The *APOE* gene is polymorphic with three major alleles, ε2, ε3 and ε4, arising from point mutations at a single gene locus to produce three common protein isoforms, ApoE2, E3, and E4. The parent form, ApoE3 has cysteine and arginine residues at positions 112 and 158, respectively, while ApoE2 (Arg158Cys) and ApoE4 (Cys112Arg) have single amino acid substitutions [15], [16]. These variant ApoE isoforms have different receptor binding activities, which affect lipoprotein clearance, while their differential affinity for triglyceride-rich lipoproteins influences lipolysis [17], [18].

In addition, ApoE has several biological functions not directly related to lipid transport, including roles in inflammation and the immune response [19], [20], which may be modulated in an isoform-dependent manner [21], [22]. Susceptibility and variable outcome of some infectious diseases is also linked to *APOE* gene polymorphism [23]–[26]. However, it remains unclear whether the plasma lipid changes induced by schistosomiasis depend on *APOE* genotype. Thus, the aim of our study was to determine whether the different *APOE* alleles influence plasma lipid levels and lipoprotein profiles in patients with hepatosplenic schistosomiasis mansoni.

## Methods

### Ethical Statement

The whole study was planned and executed following the Ethical Guidelines of the Helsinki Declaration. Participants were volunteers and all signed an informed consent statement after a full explanation about the scope of the study, including its objectives, procedures and potential risks. Ethical approval for all procedures was granted by the Human Research Ethics Committee, Center for Health Sciences, UFPE (Protocol No. 359/08).

### Study Area and Subjects

Eighty-four patients diagnosed with hepatosplenic schistosomiasis and attending the Gastroenterology Outpatient Department at the “Hospital das Clínicas - UFPE” were recruited during 2009 and 2010. The control group comprised 108 individuals with an epidemiological history incompatible with schistosomiasis and were drawn from the same age group (18–65 years) and socioeconomic background, as judged by a standardized questionnaire that enabled family budget, education level and lifestyle to be matched with those of the patients. Three stool samples from all individuals in both groups were also analyzed for parasitological infections. Subjects were excluded from the study if there was any evidence of parasitic infections, hepatitis B or C virus infections, cardiovascular or chronic kidney diseases, thyroid dysfunction or cancer. Individuals who had taken lipid-lowering drugs at anytime within the previous year were also excluded.

All participants lived in *Zona da Mata*, an endemic area in the state of Pernambuco, northeast Brazil, and their grandparents and parents were also born in this same region. The study population comprised unrelated individuals. Hepatosplenic schistosomiasis was diagnosed by physical examination and upper abdominal ultrasound, conducted by a qualified and experienced professional according to the WHO protocol for ultrasound of schistosomiasis-related morbidity [27]. The patients with hepatosplenic schistosomiasis mansoni (SM) had typical hepatosplenomegaly and portal hypertension, and at least 6 months prior to the study had been treated with praziquantel (50 mg/Kg).

### Sample Collection and Processing

Venous blood samples were drawn into evacuated tubes containing EDTA (0.562 M) after a 12 h fasting period. Plasma was separated within 2 h by centrifugation at 1500×*g* (10 min at 4°C), stored at −20°C and used for lipid analyses within 24 h. Whole blood samples were stored at 2–8°C and *APOE* genotype determined within 7 days.

### Biochemical Measurement

Plasma total cholesterol (TC) and triglyceride (TG) concentrations were assayed by routine enzymatic methods. HDL cholesterol (HDL-C) was measured after precipitation of ApoB-containing lipoproteins from plasma with phosphotungstic acid in the presence of magnesium ions [28]. Low-density lipoprotein cholesterol (LDL-C) was calculated by the Friedewald formula in subjects whose TG levels were ≤400 mg/dL [29]. Individuals whose TG levels were >400 mg/dL (six controls with the ε4-allele) were excluded from LDL-C analysis.

### Determination of *APOE* Genotype

Genomic DNA was extracted from leukocytes in whole blood, following a standard salting-out technique [30]. Single nucleotide *APOE* polymorphisms (rs7412 and rs429358) were detected by polymerase chain reaction (PCR) [31]. Amplified sequences were digested with the enzyme *Hha*I (5 units/mL) for 3 h and the restriction fragments were separated by 4% agarose gel electrophoresis and stained with ethidium bromide (0.5 mg/L).

Genotyping was performed with blinding to subject identity. Sequence-proven controls were run with each PCR. A random 1/24 of samples were genotyped again on another day; no discrepancies were observed.

### Statistical Analysis

The chi-square (χ^2^) goodness-of-fit test was used to assess deviation from Hardy-Weinberg equilibrium for each polymorphism and to compare categorical parameters among groups. All continuous variables were checked for normality and present a Gaussian distribution. Unpaired t-test was used to compare differences among continuous variables of SM patients and control individuals, while *APOE* allele groups were analyzed by one-way ANOVA followed by Fisher's Protected Least Significant Difference (PLSD). Lipid levels were adjusted for potentially confounding variables of age and gender. Pearson's Correlation test was used to estimate association between continuous parameters. Quantitative variables were expressed as mean ± standard error of media, while qualitative variables were expressed as absolute frequencies (percentage). *P*-values less than 0.05 were considered to be statistically significant. All statistical analyses were performed using StatView SAS Inc. (1998; NC, USA).

To evaluate *APOE* genotype effects on schistosomiasis mansoni, subjects were categorized into three groups: ε2 carriers (ε2/ε2+ε2/ε3 genotypes), ε3 carriers (ε3/ε3 genotype) and ε4 carriers (ε4/ε4+ε4/ε3 genotypes). In each model, the homozygous ε3/ε3 genotypes formed the reference group. Six individuals (ε2/ε4; 3.13%) were excluded from the analyses because of the putative opposing effects of these two alleles.

## Results

For this cross-sectional study, the two groups were matched by age and gender, as shown in Table 1. The frequency of *APOE* alleles among all participants were: ε2 – 11.46%, ε3 – 71.35%, and ε4 – 17.19%, similar to other studies in Brazilian populations [32]–[34]; the detailed genotype frequency is given in Table 2. All SNPs were in accordance with Hardy-Weinberg equilibrium for both SM patients (χ^2^ = 3.4164, φ = 3, p = 0.3318) and controls (χ^2^ = 3.2518, φ = 3, p = 0.3544). Both control and patient groups showed similar mean age (control: *P* = 0.3803; SM: *P* = 0.4123) and gender frequency (control: χ^2^ = 2.960, φ = 2, *P* = 0.2776; SM: χ^2^ = 2.439, φ = 2, *P* = 0.2953) among the three different alleles. The allele frequencies were not statistically different between control and SM groups (*P* = 0.3568), indicating that *APOE* polymorphism was not able to affect the chance or course of schistosomiasis in this population.

**Table 1:** Participants, genotype and lipid parameters of participants.

Parameters*	Control	SM	*P*-value
Age (years)	47.0±3.2	55.0±2.3	0.0541
Gender			
Male	30	21	-
Female	78	63	-
*N total*	108	84	0.7309
ε2	14 (13.0)	8 (9.5)	-
ε3	73 (67.6)	64 (76.2)	-
ε4	21 (19.4)	12 (14.3)	-
TC	194.4±4.5	146.4±3.0	<0.0001
LDL-C	129.0±4.5	79.8±2.7	<0.0001
HDL-C	43.4±1.4	47.9±2.7	0.0136
TG	140.6±11.9	95.8±2.8	<0.0007

SM, schistosomiasis mansoni patients; TC, total cholesterol; LDL-C, low-density lipoprotein cholesterol; HDL-C, high-density lipoprotein cholesterol; TG, triglycerides; Continuous variables are presented as mean ± standard error and were compared by unpaired t-test, whereas categorical variables are presented as absolute (percentage) frequencies and were compared by the Chi-square test.

*Plasma lipids are expressed in mg/dL.

**Table 2:** *APOE* genotype frequencies among patients with hepatosplenic schistosomiasis mansoni and controls.

Genotype	Control	SM
ε2/ε2	2	0
ε2/ε3	12	8
ε2/ε4	2	4
ε3/ε3	73	64
ε3/ε4	18	11
ε4/ε4	3	1
Total	108	84

When compared to healthy controls, the SM patients showed significant reductions (*P*<0.0001) in the plasma levels of TC (25%), LDL-C (38%) and TG (32%). By contrast, the concentration of HDL-C was significantly increased in the patients (10% higher; *P* = 0.0136) (Table 1).

**Figure 1:**
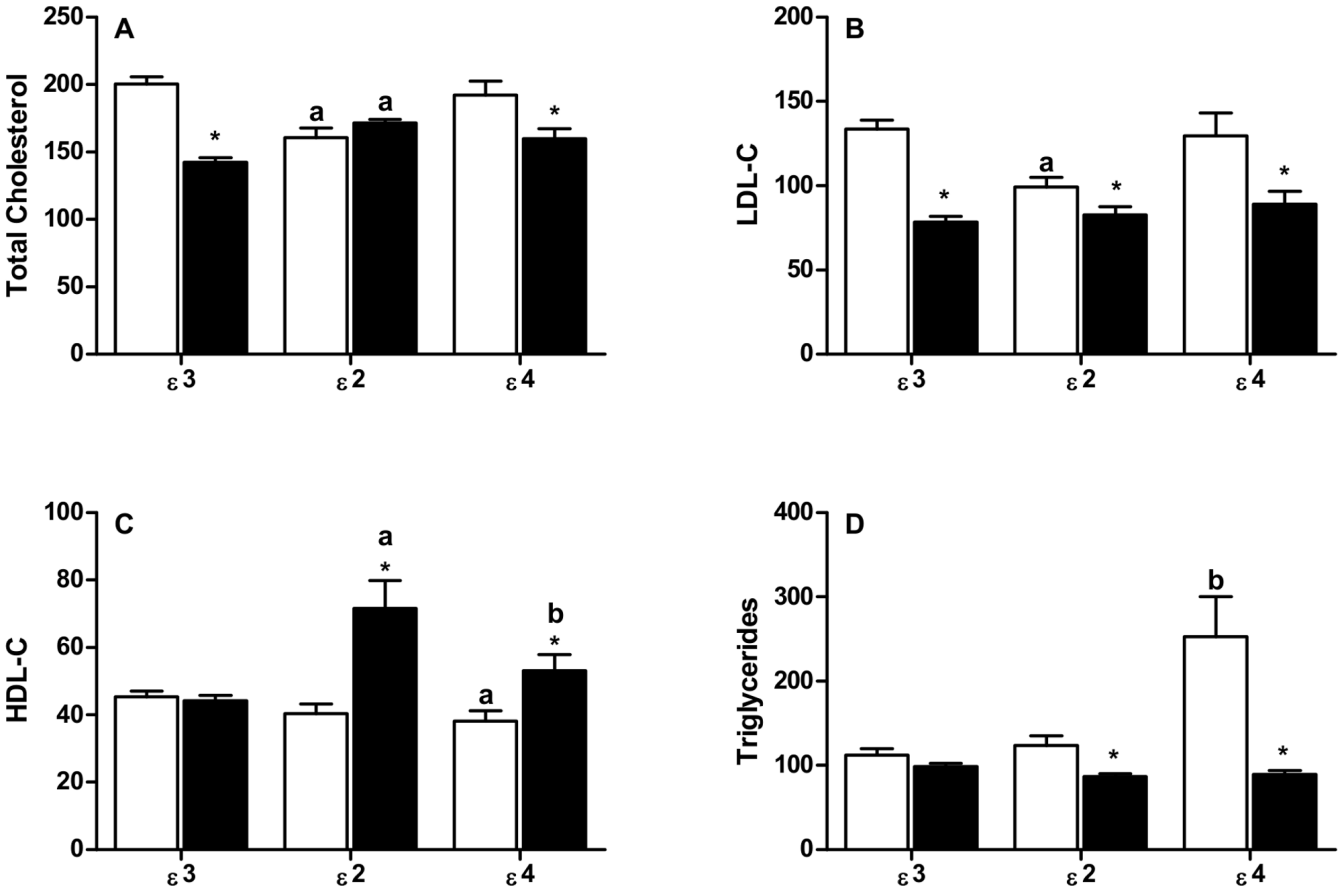
Effect of *APOE* gene polymorphism on plasma levels of Total Cholesterol (A), LDL-C (B), HDL-C (C), and Triglycerides (D) in Control subjects (open bars) and SM patients (filled bars). Plasma lipids are expressed in mg/dL. *  =  *P*<0.05 for group of SM patients vs. Control of the same allele. Comparisons between the different alleles of the same SM patient group, or of the same Control group, are indicated as follows: **a**  = *P*<0.05 vs. ε3; **b**  = *P*<0.05 vs. ε2 and ε3. Exact *P* values are given in Table 3.

To assess the influence of *APOE* gene polymorphism on plasma lipid parameters, we repeated the analyses after subdividing each group on the basis of *APOE* alleles. Lower TC and LDL-C levels were found in the ε3 subgroup (ε3/ε3 genotype) of SM patients, as observed without allele differentiation (Figure 1A and 1B). However, the increases in HDL-C and reductions in TG noted for all SM patients were not seen, even though the ε3/ε3 genotype is carried by 70% of participants (Figure 1C and D). This analysis of the ε3/ε3 genotype allows the influence of schistosomiasis on human lipid metabolism to be evaluated without possible interfering factors from the inclusion of ε2 and ε4 alleles. All the values of *P* from comparisons showed in Figure 1 are shown in Table 3.

**Table 3:** Values of *P* from comparisons showed in Figure 1.

		TC	LDL-C	HDL-C	TG
Control vs SM	ε3	<0.0001	<0.0001	0.6337	0.1422
	ε2	0.8494	0.0098	0.0005	0.0166
	ε4	0.0403	0.0219	0.0113	0.0152
Control	ε2 vs ε3	0.0052	0.0157	0.2533	0.7348
	ε2 vs ε4	0.0547	0.0842	0.6825	0.0018
	ε3 vs ε4	0.4590	0.7536	0.0431	<0.0001
SM	ε2 vs ε3	0.0458	0.9933	<0.0001	0.3366
	ε2 vs ε4	0.8342	0.3445	0.0316	0.9945
	ε3 vs ε4	0.0586	0.1883	0.0413	0.2727

ANOVA followed by Fisher's PLSD test.

Despite this significantly lower plasma TC in ε3 patients, the TC levels were similar (*P* = 0.5360) in ε2 patient and control groups. This reflected a marked HDL-C increase (77%) and a positive correlation between TC and HDL-C (R = 0.724; *P* = 0.0250) for patient ε2-carriers. By contrast, LDL-C was reduced in ε2 patients (as it was in ε3 patients; Figure 1A) and unrelated to TC levels (R = 0.225; *P* = 0.5750).

Plasma cholesterol changes associated with schistosomiasis were also noted for ε4-carriers. As with the ε2 allele, the ε4 SM patients had increased HDL-C (39% higher) compared to their control counterparts (Figure 1C); and like ε3-carriers they had reduced TC and LDL-C (Figure 1A and 1B).

Decreased plasma TG concentrations were seen in SM patients with the ε2 or ε4 variant alleles, but not for the ε3/ε3 genotype. However, the most striking difference in TG was noted in healthy ε4-carriers; their TG concentration was two-fold higher than the five other subgroups (Figure 1D).

## Discussion

Our report is the first to identify a host genetic factor, *APOE* polymorphism, which influences the extent and nature of plasma lipid changes associated with schistosomiasis mansoni. In future studies, this finding will help in understanding how the parasite affects particular steps in host lipid metabolism and how host genetic background modifies disease progression and morbidity.

Several studies have shown the *APOE* genotype to influence infection susceptibility and damage in certain diseases caused by viruses, including human immunodeficiency virus [18] and hepatitis C [26], [35] and B [36], protozoa [23] and fungi [24]. As allele frequencies were similar for patients and controls, we infer that the different ApoE isoforms do not affect progression of schistosomiasis to the chronic hepatosplenic condition. Conceivably, this conclusion may not hold for the earlier, less severe hepatointestinal stage or for hepatosplenic patients subdivided on the extent of liver fibrosis [3]. Though of interest, as *APOE* alleles are suggested to affect fibrosis progression in hepatitis C infection [26], [37], a much larger patient population would be needed to ensure adequate power for subtle genotype effects [35]. Gene studies of individuals infected with schistosomiasis have found significant associations of cytokines related to the immune response [38]–[40]. However, to date and similar to our result, no study has reported a link between the *APOE* gene polymorphism and schistosomiasis prevalence or severity.

The changes we report in plasma lipoprotein profiles, reductions in TC and LDL-C and an increase in HDL-C, is considered cardioprotective and hence can be regarded as a beneficial side-effect of schistosomiasis. We, and others, have previously reported low plasma total cholesterol in human studies [6], [7] and in infected animals [11]–[13], [41]. Doenhoff et al. [11] have shown that when fat-fed ApoE-deficient mice are infected with *S. mansoni* the decrease in plasma cholesterol is associated with a 50% reduction in atherosclerotic plaque progression, consistent with the low frequency of atherosclerosis noted in schistosomiasis patients [6], [42].

Schistosomes need, but do not synthesize, cholesterol and one explanation for reduced host plasma cholesterol is that adult worms internalize LDL, via tegumental proteins analagous to mammalian LDL receptors [43]. Another suggestion is that the worms shed antigenic glycosyl-phosphatidylinositol (GPI)-anchored proteins into the circulation, which are sequestered by host lipoprotein particles [44]. Subsequent, antibody attack leads to lipoprotein removal by neutrophil endocytosis, although any plasma cholesterol-lowering effect in vivo has yet to be assessed. Against both these mechanisms is the failure of same-sex worms to lower cholesterol during mouse infections [41], implying that adult worms alone are not responsible and that the parasite's eggs are hypocholesterolemic. This concept is supported by La Flamme et al. [12] who noted reduced plasma cholesterol in mice chronically exposed to schistosome eggs, while Stanley et al. [41] found that soluble factors released from *S. mansoni* eggs were responsible.

Although TC and LDL-C were decreased in SM patients, we noted increased levels of HDL-C, consistent with an early report that alpha-lipoproteins were significantly higher in patients with Bilharzial hepatic fibrosis [6]. By contrast, infection of ApoE-deficient mice with *S. mansoni* cercariae resulted in reduced HDL-C [11], although levels do not change during chronic exposure to schistosome eggs [12]. However, direct comparisons of mouse and human plasma lipoprotein metabolism are complex, as there is a marked difference in the LDL-C to HDL-C ratio [13]. Absence of cholesteryl ester transfer protein (CETP) in mice increases HDL levels compared to humans [45], while much of their LDL is cleared rapidly using ApoE as ligand rather than the slow ApoB100 pathway used by human LDL [46].

Effects of infection and inflammation on host lipoprotein metabolism are multi-faceted. Although the acute-phase response inhibits ApoAI synthesis and lowers HDL-C [47], [48], the most profound changes are in structure and composition, which transform the HDL from anti-inflammatory to proinflammatory particles [49]–[51]. Such pathological changes in HDL have yet to be studied in hepatosplenic schistosomiasis, although chronic inflammation is known to impair reverse cholesterol transport and the antioxidant capacity of HDL [52]–[54]. Indirect evidence suggests that HDL in schistosomiasis patients is a poor antioxidant, as we previously found elevated levels of erythrocyte lipid peroxidation [55].

In humans, *APOE* polymorphism is well-documented to affect plasma TC and LDL-C; for example, meta-analyses by Bennet et al. [56] found differences between ε2/ε3 and ε3/ε4 carriers, the most common genotypes after ε3/ε3, of 8% and 14%, respectively. As indicated earlier, differential binding affinities of the individual ApoE isoforms to receptors and for surfaces of triglyceride-rich lipoprotein particles underlie such variation [16], [18]. We also noted effects of *APOE* genotype on TC and LDL-C in our controls as mean values were significantly lower in ε2-carriers compared to the ε3/ε3 group (*P* = 0.0052 and *P* = 0.0157, respectively), though unchanged for the ε4-allele. Interestingly, schistosomiasis abolished, and indeed reversed, this relationship; ε2 patients had higher mean TC and LDL-C than ε3/ε3 patients, although only the TC difference reached significance (*P* = 0.0458).

The relation of *APOE* genotypes with HDL-C was reported by Bennet et al. [56] to be inverse and weak with a 5% difference between the ε2/ε3 and ε3/ε4 carriers. Despite small numbers, we also noted a slight but significant fall of HDL-C in control ε4 carriers compared to their ε3/ε3 counterparts. The small (10%) HDL-C increase in our schistosomiasis patients (Table 1) was due to higher levels in ε2- and ε4-carriers, as the HDL-C of ε3/ε3 genotypes was near-identical for controls and patients. Can these findings be explained? One difficulty is the complexity of HDL formation, maturation and clearance, namely reverse cholesterol transport, which though involving the major HDL protein, ApoAI, is influenced and assisted at each step by ApoE [57]. Thus, initial sequestration of excess cellular cholesterol [58], activation of the cholesterol esterifying enzyme, plasma lecithin-cholesterol acyltransferase (LCAT) [59] and cholesterol ester delivery to the liver [17], [60] are all processes that involve ApoE in an isoform-dependent manner. To this complexity, we can overlay HDL metabolic changes due to *S. mansoni* infection and associated inflammatory responses and fibrogenesis. For example, we have reported LCAT deficiency in human [7] and animal [13] schistosomiasis, while decreased CETP activity is a feature of the acute-phase response [47] and raises HDL levels, particularly ApoE-rich HDL [18], [61].

We can speculate, therefore, that HDL-C increases in schistosomiasis are a multi-step process, promoted by low CETP activity and enhanced further by the ε2-allele: ApoE2 has a higher affinity than ApoE3 or ApoE4 for HDL [62], allowing the particles to expand in size [18], [63], while defective ApoE2 receptor binding delays their clearance from plasma via hepatic LDL-receptors [17], [60]. A different scenario is required to explain the HDL-C increase in the ε4-carrying patients, since ApoE4 associates poorly with HDL and has high affinity for LDL receptors, properties predicted to reduce HDL-C. One tentative possibility is that the poor antioxidant capacity of cysteine-negative ApoE4 [64] allows excessive formation of oxidized HDL, particularly as *S. mansoni* infections markedly increase oxidative stresses [65]. As oxidized HDL impedes normal reverse cholesterol transport [51]–[53], this delay in maturation may increase HDL-C in patients with ε4-alleles.

The plasma triglycerides change has inconsistent results in humans, as seen in mice earlier reports [9], [11], [12]. We observed 30% reduction in plasma triglycerides in SM patients. The mechanism(s) causing reduced plasma TG is uncertain. It may simply reflect lower levels of non-HDL lipoproteins since the acquired LCAT deficiency of human schistosomiasis increases the TG:CE ratio of core lipids [9], or be independent of infection-related responses, as pulmonary fibrosis with non-infectious origins results in low plasma TG [64]. A direct effect is also possible, as *S. mansoni* infected mice had reduced hepatic expression of acetyl coenzyme A acyltransferase, an enzyme involved in fatty acid metabolism [67]. Nevertheless, data from animal studies are inconsistent. Infection of non-human primates resulted in TG rises >10% after 30 or 60 days, whereas in mice TG levels were reported to rise two-fold [11] or be unchanged 7–10 weeks post-infection [66], or to significantly decline from the 4^th^ week [68].

Meta-analyses to assess association of *APOE* genotypes with plasma triglycerides report non-linear relationships, the ε2- and ε4-carriers having higher levels than those with the ε3/ε3 genotype [56], [69], [70]. For ApoE2 the simplest explanation is reduced binding and delayed hepatic clearance of VLDL remnants, whereas a dual mechanism is invoked for ApoE4; impaired lipolysis because ApoE4 has higher affinity for VLDL [17] and, paradoxically as it has high receptor binding, by the failure of ApoE4 to accelerate hepatic removal of VLDL remnants due to inefficient recycling of the ApoE4 protein into the Space of Disse [71]. Consistent with the data from meta-analyses [56], [69], [70], we found that the mean TG values for control individuals with ε4- and ε2-alleles were higher than the ε3/ε3 group (though *P*<0.05 only for ε4-carriers; Figure 1D). Unexpectedly, the mean TG (252±48 mg/dL) of this ε4 control group was much higher than seen in other studies, including different Brazilian populations [30]–[32], [72], an unexpected finding for which we have no immediate explanation. Of greater interest, however, was that the mean TG level in the ε3/ε3 patients was higher, albeit not significant, than levels in patients carrying ε2- or ε4-alleles (Figure 1D). These data suggest that the mechanism(s) which promotes increased plasma TG in healthy ε2- and ε4-carriers is either inoperative or ineffective in schistosomiasis patients.

One limitation of this study is that it was conducted only at a single hospital, the Hospital das Clinicas, UFPE, which is the reference hospital for schistosomiasis in Pernambuco State, Brazil. Here, the Gastroenterology Outpatient Department receives the most severe cases of schistosomiasis, usually patients with a history of one or more episodes of gastrointestinal bleeding and hence most of the patients have the hepatosplenic form of the disease. Moreover, we had no information on plasma lipid levels before infection to compare with levels after the patients had developed the hepatosplenic form of schistosomiais. Therefore, the findings from the present study may not be extrapolated to all patients from other endemic areas who present with the hepatosplenic form of the disease.

In summary, we confirm that human schistosomiasis causes dyslipidemia and, for the first time, report that certain changes in plasma lipid levels and lipoprotein profiles are dependent on patient *APOE* gene polymorphism. Importantly, we also conclude that the normal regulation of plasma lipid levels by *APOE* genotype is disrupted by schistosomiasis mansoni. This finding merits further investigation; it may uncover new metabolic pathways and pathological processes associated with human schistosomiasis. In turn, these may identify molecular targets to aid treatment of schistosomiasis morbidity, and perhaps also inform other lipid-associated diseases, including atherosclerosis and diabetes.
